# Comparing diagnostic performance of Cantonese-Chinese version of Rome IV criteria and a short Reference Standard for functional dyspepsia in China

**DOI:** 10.1186/s12876-022-02520-6

**Published:** 2022-10-12

**Authors:** Leonard Ho, Shuijiao Chen, Fai Fai Ho, Charlene H. L. Wong, Jessica Y. L. Ching, Pui Kuan Cheong, Irene X. Y. Wu, Xiaowei Liu, Ting Hung Leung, Justin C. Y. Wu, Vincent C. H. Chung

**Affiliations:** 1 School of Chinese Medicine, Faculty of Medicine, The Chinese University of Hong Kong, Shatin, Hong Kong; 2 Department of Gastroenterology, Xiangya Hospital, 110 Xiangya Road, Kaifu District, Changsha, Hunan China; 3 Hunan International Scientific and Technological Cooperation Base of Artificial Intelligence Computer-Aided Diagnosis and Treatment for Digestive Disease, Changsha, Hunan China; 4 The Jockey Club School of Public Health and Primary Care, Faculty of Medicine, The Chinese University of Hong Kong, Shatin, Hong Kong; 5 Institute of Digestive Diseases, The Chinese University of Hong Kong, Shatin, Hong Kong; 6 Xiangya School of Public Health, Central South University, 238 Shang Ma Yuan ling Alley, Kaifu District, Changsha, Hunan China; 7 Department of Medicine and Therapeutics, Faculty of Medicine, The Chinese University of Hong Kong, Shatin, Hong Kong

**Keywords:** Dyspepsia, Gastrointestinal diseases, Validation study, Sensitivity and specificity

## Abstract

**Introduction:**

Functional dyspepsia (FD) is diagnosed based on self-reported symptoms and negative upper gastrointestinal endoscopic findings. The Rome criteria were not adopted as a diagnostic instrument in clinical guidelines due to their complexity. Different guidelines used relatively simple symptom assessment schemes with contents that vary significantly. A previously evaluated short Reference Standard may serve as a more standardised tool for guidelines. We evaluated its diagnostic accuracy against the Rome IV criteria in a cross-sectional study in Hong Kong.

**Methods:**

A total of 220 dyspeptic patients sampled consecutively from a tertiary hospital and the community completed the Rome IV diagnostic questionnaire, which was translated into Cantonese-Chinese, and the Reference Standard. Sensitivity, specificity, positive and negative likelihood ratios (LRs), and area under the receiver operating characteristics curve (AUC), with 95% confidence intervals (CIs), were calculated.

**Results:**

Among the participants, 160 (72.7%) fulfilled the Reference Standard with negative upper gastrointestinal endoscopic results. The Reference Standard identified patients with Rome IV-defined FD with 91.1% (95% CI 82.6%–96.4%) sensitivity and 37.6% (95% CI 29.6%–46.1%) specificity. The positive and negative LRs were 1.46 (95% CI 1.26–1.69) and 0.24 (95% CI 0.11–0.49), respectively. The AUC value was 0.64 (95% CI 0.59–0.69).

**Conclusions:**

The Reference Standard can rule out patients without Rome IV-defined FD. It may be used as an initial screening tool for FD in settings where the use of the Rome IV criteria is impractical. It may also provide a uniform definition and diagnostic rule for future updates of clinical guidelines.

**Supplementary Information:**

The online version contains supplementary material available at 10.1186/s12876-022-02520-6.

## Introduction

Functional dyspepsia (FD) is a functional gastrointestinal (GI) disorder characterised by postprandial fullness, early satiation, epigastric pain, or epigastric burning that is unexplainable by routine investigations [1]. Patients with dominant postprandial fullness or early satiation are categorised into the symptom subtype of postprandial distress syndrome (PDS), while those with dominant epigastric pain or epigastric burning into epigastric pain syndrome (EPS) [1]. An overlap of symptom subtypes is also common in routine practice [2]. FD has a prevalence of 10–40% among populations in the West, while in Asia the prevalence has a lower range of 5–30% [2]. It impacts patients’ work productivity and health-related quality of life, apart from being a financial burden on patients and health systems [3, 4].

FD and other functional GI disorders are gut-brain interaction disorders, of which diagnosis is primarily based on symptoms that may not be justified by corresponding physiological evidence [5]. The Rome diagnostic criteria have been developed to systematise the definition, epidemiology, pathophysiology, and management of functional GI disorders [6, 7]. Relevant Rome diagnostic questionnaires have also been derived to serve as standardised inclusion criteria for clinical research [5]. The latest Rome IV criteria define FD as the presence of PDS and/or EPS in the past three months, with symptom onset six months before diagnosis, and without evidence of organic upper GI disease that may explain the symptoms [1]. The full definition is illustrated in Table 1. Although the Rome IV FD diagnostic questionnaire was published five years ago, it is yet to be translated into Cantonese-Chinese (Cantonese). There is a demand for a Cantonese version of the Rome IV FD diagnostic questionnaire as it is the dominant language in the China Greater Bay Area and among overseas Chinese communities. The diagnostic performance of the translated version will also require evaluation.

**Table 1:** Definitions of functional dyspepsia according to the Rome IV criteria and the Reference Standard

	Rome IV criteria (Rome IV-defined FD)^1^	Reference Standard^12^
General definition	In the past three months, with symptom onset six months before diagnosis: Rome IV-defined PDS; **AND/OR** Rome IV-defined EPS	In the past month, with one of the following symptoms (at least once per week): Postprandial fullness; Early satiety; Epigastric pain; **OR** Epigastric burning
	**AND** No evidence of organic upper gastrointestinal disease that is likely to explain the symptoms	
Postprandial distress syndrome (PDS)	At least three days per week: Bothersome postprandial fullness; **OR** Bothersome early satiety	At least once per week: Any degree of postprandial fullness; **OR** Any degree of early satiety
Epigastric pain syndrome (EPS)	At least one days per week: Bothersome epigastric pain; **OR** Bothersome epigastric burning	At least once per week: Any degree of epigastric pain; **OR** Any degree of epigastric burning

FD, Functional dyspepsia

Due to the lack of feasibility in using the lengthy Rome diagnostic criteria in routine practice, diagnostic definitions of FD documented in recent clinical guidelines did not adhere to any editions of the Rome criteria. As illustrated in Table 2, the diagnostic criteria reported in different guidelines are inconsistent [8–11]. The lack of a consistent definition across guidelines contributes to the uncertainty on who should be diagnosed with FD and require initiation of treatment.

**Table 2:** Definitions of functional dyspepsia according to the Asian, North American, Korean, and European clinical guidelines

	2012 Asian Consensus Report on FD^8^	2017 ACG/CAG Clinical Guideline for FD^9^	2020 Clinical Practice Guideline for FD in Korea^10^	2021 UEG/ESNM Consensus on FD^11^
Definition of FD	In the past three months, with one of the following symptoms: Postprandial fullness; Early satiety; Epigastric pain; Epigastric burning; Bloating in the upper abdomen; Nausea; Vomiting; **OR** Belching	In the past month, the patient must present with predominant epigastric pain. Predominant epigastric pain may be associated with any other upper gastrointestinal symptoms, such as epigastric fullness, nausea, vomiting, or heartburn	In the past month, the patient must present with one of the following symptoms: Pain or discomfort in the upper abdomen; Postprandial fullness; Early satiety; Bloating; Nausea; **OR** Vomiting	The patient must present with one of the following symptoms (no recall period specified): Postprandial fullness; Early satiety; Epigastric pain; **OR** Epigastric burning
	**AND** No evidence of organic upper gastrointestinal disease that is likely to explain the symptoms			

ACG, American College of Gastroenterology; CAG, Canadian Association of Gastroenterology; ESNM, European Society for Neurogastroenterology and Motility; FD, Functional dyspepsia; UEG, United European Gastroenterology

The use of concise diagnostic instruments, such as the Reference Standard developed by Ford et al. [12], may be favoured in routine practice as it can be administered easily as a self-reported questionnaire. The Reference Standard is one of the clinically accepted questionnaires for diagnosing FD, and indeed it has previously been used as the diagnostic gold standard in validation studies of the Rome criteria [12, 13]. It defines FD as the presence of any postprandial fullness, early satiety, epigastric pain, or epigastric burning at least once per week in the past month, without evidence of organic upper GI disease that may explain the symptoms (Table 1). Upon confirming its diagnostic performance, the Reference Standard may be adopted in future clinical guidelines as the diagnostic criterion for FD. This will provide the field with a more uniform diagnostic rule from a practical perspective.

In this study, we translated the Rome IV FD Diagnostic Questionnaire (R4DQ-FD) into Cantonese and evaluated the diagnostic accuracy of the Cantonese Reference Standard via a cross-sectional study in Hong Kong. The Cantonese R4DQ-FD was selected as the gold standard to be compared against the index test of Reference Standard [12]. We hypothesised that the Reference Standard is a sufficient proxy for the R4DQ-FD in terms of diagnostic performance in identifying FD patients in routine practice.

## Methods

### Phase one: translating the Rome IV diagnostic questionnaire for functional dyspepsia

Before the translation process, official approval was sought from the Rome Foundation for the use and translation of R4DQ-FD. The official English version of the R4DQ-FD were translated to Cantonese by professional medical translators using the five-step forward–backward translation method [14] and according to the guidelines prescribed by the Rome Foundation [15]. The Cantonese R4DQ-FD were then pre-tested in ten FD patients before data collection. Details of the translation process are illustrated in Additional file 1: Appendix 1.

### Phase two: assessing diagnostic performance of Reference Standard

#### Participants and setting

The cross-sectional study was carried out from December 2020 to April 2021. According to the formula developed by Jones et al. [16], a sample size of 220 subjects is needed for estimating the diagnostic performance indicators, if the confidence levels, the sensitivity of the Reference Standard, and the prevalence of FD were assumed to be 5%, 95.5%, and 30% respectively. Following the QUADAS-2 (Quality Assessment of Diagnostic Accuracy Studies 2) tool recommendation on patient recruitment [17], we sampled dyspeptic patients consecutively from GI outpatient department of the Hong Kong Prince of Wales Hospital and the community. Both consecutive sampling and random sampling are recommended for diagnostic studies [17], yet an overview of reviews illustrated that studies with non-consecutive sampling of participants might have significantly higher estimates of diagnostic accuracy [18]. Therefore, we implemented consecutive sampling in this study.

The Prince of Wales Hospital is the leading public hospital in the New Territories East Cluster, the largest cluster in the Hong Kong Hospital Authority in terms of geographic coverage [19]. During 2020 and 2021, the hospital had 766,049 specialist outpatient attendances, constituting more than 10% of the total number of specialist outpatient attendances in Hong Kong, including GI consultations [20, 21].

The eligibility of potential subjects was confirmed using the following inclusion and exclusion criteria:

Inclusion criteria—(1) completed oesophagogastroduodenoscopy (OGD) within five years; (2) tested for Helicobacter pylori (H. pylori) within five years; (3) age of at least 18 years; AND (4) willing to provide written or verbal informed consent.

Exclusion criteria—(1) without an accessible OGD report; (2) without an accessible test result for H. pylori; OR (3) does not understand written and verbal Cantonese.

OGD reports and H. pylori test results were retrieved from electronic health records or reports provided by potential subjects. Ethical approval was obtained from the Joint Chinese University of Hong Kong—New Territories East Cluster Clinical Research Ethics Committee (Reference number: CREC 2018.325) and the Survey and Behavioural Research Ethics Committee of the Chinese University of Hong Kong (Reference number: SBRE-20-093) before study commencement.

### Data collection

#### Basic characteristics and measurements

After obtaining written informed consent, recruited patients were invited to complete a structured, self-administered online questionnaire once their eligibility was confirmed. The questionnaire consisted of five parts: (1) sociodemographic characteristics; (2) self-assessed health status and co-morbidities; (3) drinking and smoking habits; (4) Cantonese version R4DQ-FD; and (5) Reference Standard for FD. Self-assessed health status is widely used in different settings and research fields to measure participants’ overall health at a given point in time [22, 23]. It consists of one question on a five-point Likert scale: *“In general, would you consider your health is excellent, good, fair, poor, or very poor?”*.

### Organic upper gastrointestinal diseases that are likely to explain the symptoms

An organic upper GI disease referred to pathologies located in the upper GI tract, identifiable by conventional diagnostic procedures, and is likely to cause improvement or resolution of dyspeptic symptoms when it is improved or eliminated [1, 12, 24]. Examples include, but are not limited to, erosive or reflux oesophagitis, Barrett’s oesophagus, oesophageal carcinoma, gastric ulcer, and H. Pylori-associated gastritis. Patients with any organic upper GI diseases that were likely to explain their dyspeptic symptoms as indicated by the OGD reports were not classified as having Reference Standard or Rome IV-defined FD.

### Data analysis

#### Characteristics of patients

Sociodemographic and health-related characteristics of the recruited patients were summarised and presented in appropriate descriptive statistics, including frequency, percentage, mean, and standard deviation (SD). Using Fisher exact tests, the prevalence of organic diseases in patients who met the Reference Standard for FD was compared with those who did not. Similar comparisons were made between different FD symptom subtypes. A p-value < 0.050 was considered statistically significant. The above statistical analyses were performed using Microsoft Excel 2016.

#### Evaluation of the performance of the Reference Standard for functional dyspepsia

Performance of the Reference Standard as an index test in identifying patients with Rome IV-defined gold standard was assessed by computing the diagnostic performance indicators [25]. Diagnostic performance of PDS and EPS modules of the Reference Standard were also assessed. The sensitivity, specificity, likelihood ratios (LRs), predictive values, and the area under the receiver operating characteristics curve (AUC) along with their 95% confidence interval (CI) were calculated using IBM SPSS Statistics 28. Formulae for the calculations are shown in Additional file 1: Appendix 2.

## Results

### Characteristics of recruited patients

**Fig. 1:**
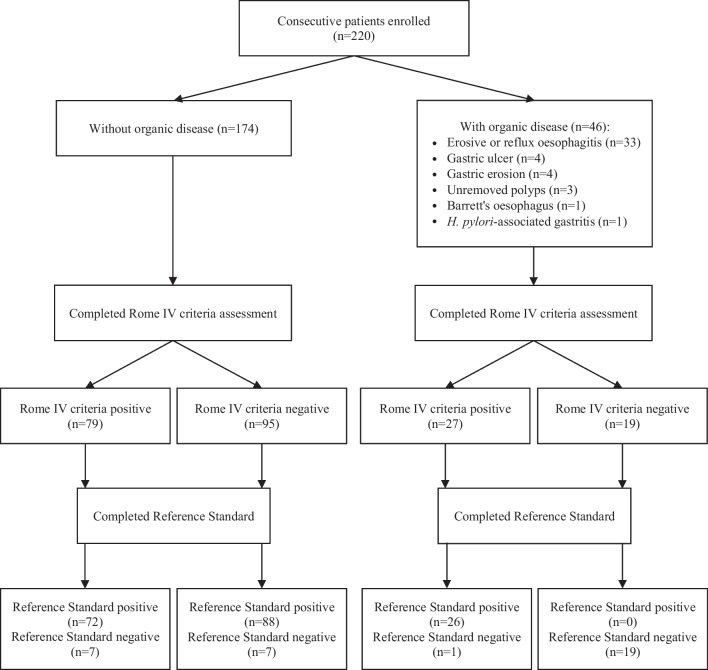
Flow of study participants

We approached a total of 749 subjects from the GI outpatient department of the Prince of Wales Hospital and the community, with 456 agreeing to participate. One-hundred-and-thirty-seven failed to fulfil the inclusion criteria due to missing valid medical reports (n = 97) and absence of dyspeptic symptoms (n = 40). Eventually, we received 220 completed questionnaires among the remaining 319 participants (Fig. 1). The response rate was 69.0%. No follow-up was needed for this diagnostic study.

Their mean age was 52.7 years (SD: 12.8), and 117 (80.4%) of them were female. The mean body mass index was 22.0 (SD: 3.8). Twelve (5.4%) and sixty (27.2%) patients were regular users of tobacco and alcohol, respectively. Although 156 (70.9%) of them reported having no other chronic conditions, 154 (70.0%) reported that their health status was poor or very poor. Regardless of the presence of organic upper GI diseases as indicated by medical records, 186 (84.5%) and 106 (48.2%) patients fulfilled the Reference Standard and the Rome IV criteria for FD, respectively. Details on the sociodemographic and health-related characteristics are reported in Table 3.

**Table 3:** Sociodemographic and health-related characteristics of 220 patients

Sociodemographic and health-related characteristics	Results
Mean age, year (SD)	52.7 (12.8)
Mean body mass index, kg/m^2^ (SD)	22.0 (3.8)
Female sex, *n* (%)	177 (80.4)
Tobacco user, *n* (%)	12 (5.4)
Alcohol user, *n* (%)	60 (27.2)
*Educational level, n (*%*)*	
Primary school	19 (8.6)
Junior Secondary (Secondary 1–3)	55 (25.0)
Senior Secondary (Secondary 4–5)	56 (25.5)
Sixth form, industrial training, or vocational training	15 (6.8)
College (non-degree programme or associate degree)	13 (5.9)
University	62 (28.2)
*Occupation, n (%)*	
Retired	44 (20.0)
Unemployed	7 (3.2)
Student	4 (1.8)
Homemaker	31 (14.1)
Employed	134 (60.9)
*Number of chronic conditions, n (%)*	
None	156 (70.9)
1	41 (18.6)
2	14 (6.4)
≥ 3	9 (4.1)
*Self-assessment of health status, n (%)*	
Excellent	0 (0.0)
Good	12 (5.5)
Fair	54 (24.5)
Poor	128 (58.2)
Very poor	26 (11.8)
*Met Reference Standard for FD, n (%)*	
Total	186 (84.5)
Patients without organic upper GI diseases	160 (72.7)
*Met Rome IV criteria for FD, n (%)*	
Total	106 (48.2)
Patients without organic upper GI diseases	79 (35.9)

FD: Functional dyspepsia; GI: Gastrointestinal; SD: Standard deviation

### Prevalence of organic diseases

At least one organic upper GI disease was detected in twenty-six (14.0%) of the 186 patients who met the Reference Standard for FD, with erosive or reflux oesophagitis being the most common diagnosis (Table 4). Gastric erosion and gastric ulcer were also diagnosed in 2.1% of the sample. Likewise, erosive or reflux oesophagitis was the most prevalent organic upper GI finding in those who did not meet the Reference Standard. Barrett's oesophagus, gastric erosion, H. Pylori-associated gastritis, gastric ulcer, and unremoved polyps were found in 19.5% of the sample. There was a significant difference between patients who met the Reference Standard and those who did not in the prevalence of erosive or reflux oesophagitis (11.8% for patients meeting the criteria versus 32.4% for those not; *p* = 0.003), gastric ulcer (0.5% for patients meeting the criteria versus 8.8% for those not; *p* = 0.010), and unremoved polyps (0.0% for patients meeting the criteria versus 8.8% for those not; *p* = 0.003).

**Table 4:** Prevalence of organic diseases in patients meeting the questionnaire part of the Reference Standard for functional dyspepsia, compared with those who did not

Organic diseases	Met Reference Standard for FD (n = 186, questionnaire only)	Did not met Reference Standard for FD (n = 34, questionnaire only)	*p* values*
Erosive or reflux oesophagitis, *n* (%)	22 (11.8)	11 (32.4)	0.003
Barrett's oesophagus, *n* (%)	0 (0.0)	1 (2.9)	0.147
Gastric erosion, *n* (%)	3 (1.6)	1 (2.9)	0.485
*H. pylori*-associated gastritis, *n* (%)	0 (0.0)	1 (2.9)	0.148
Gastric ulcer, *n* (%)	1 (0.5)	3 (8.8)	0.010
Unremoved polyps, *n* (%)	0 (0.0)	3 (8.8)	0.003

FD, Functional dyspepsia; *H. pylori*, Helicobacter pylori

^*^p value for Fisher exact tests for comparisons of categorical data; < 0.050 was considered statistically significant

Of the 186 patients meeting the Reference Standard for FD, sixty-three (33.9%) met the criteria for PDS alone, fourteen (7.5%) met the criteria for EPS alone, and 109 (58.6%) met the criteria for both PDS and EPS (Table 5). Erosive or reflux oesophagitis was the dominant organic disease among the symptom subtypes. Significant difference was detected in the prevalence of erosive or reflux oesophagitis between the three groups of patients (9.5% for PDS patients versus 35.7% for EPS patients versus 8.3% for patients with both PDS and EPS; *p* = 0.018).

**Table 5:** Prevalence of organic diseases in patients meeting the questionnaire part of the Reference Standard for functional dyspepsia, according to symptom subtype

Organic diseases	Met criteria for PDS alone (n = 63, questionnaire only)	Met criteria for EPS alone (n = 14, questionnaire only)	Met criteria for PDS and EPS (n = 109, questionnaire only)	*p* values*
Erosive or reflux oesophagitis, *n* (%)	6 (9.5)	5 (35.7)	9 (8.3)	0.018
Gastric erosion, *n* (%)	1 (1.6)	0 (0.0)	2 (1.8)	1.000
Gastric ulcer, *n* (%)	1 (1.6)	0 (0.0)	0 (0.0)	0.409

EPS, Epigastric pain syndrome; PDS, Postprandial distress syndrome

*p value for Fisher exact tests for comparisons of categorical data; < 0.050 was considered statistically significant

### Diagnostic performance of the Reference Standard

Among the seventy-nine patients with FD according to the Rome IV criteria, seventy-two met the Reference Standard for FD (Table 6), illustrating a sensitivity of 91.1% (95% CI 82.6%–96.4%) (Table 7). In the 141 patients who were not judged to have Rome IV-defined FD, fifty-three did not meet the Reference Standard, illustrating a specificity of 37.6% (95% CI 929.6%–46.1%). The positive LR of the Reference Standard for FD was 1.46 (95% CI 1.26–1.69), and the negative LR was 0.24 (95% CI 0.11–0.49). The AUC for the Reference Standard was 0.64 (95% CI 0.59–0.69).

**Table 6:** Cross tabulation of the Reference Standard results by the Rome IV criteria results

	Rome IV criteria		
	Positive	Negative	Total
(a) *Results for functional dyspepsia*			
Reference Standard			
Positive	72	88	160
Negative	7	53	60
Total	79	141	220
(b) *Results for postprandial distress syndrome*			
Reference Standard			
Positive	63	88	151
Negative	3	66	69
Total	66	154	220
(c) *Results for epigastric pain syndrome*			
Reference Standard			
Positive	33	72	105
Negative	9	106	115
Total	42	178	220

**Table 7:** Sensitivity, specificity, positive and negative likelihood ratios, and positive and negative predictive values for the Reference Standard

	Sensitivity (95% CI)	Specificity (95% CI)	Positive likelihood ratio (95% CI)	Negative likelihood ratio (95% CI)	Positive predictive value (95% CI)	Negative predictive value (95% CI)
Reference Standard for FD	91.1% (82.6%–96.4%)	37.6% (29.6%–46.1%)	1.46 (1.26–1.69)	0.24 (0.11–0.49)	45.0% (36.3%–67.8%)	88.3% (77.7%–91.5%)
Reference Standard for PDS	95.5% (87.3%–99.1%)	42.9% (34.9%–51.1%)	1.67 (1.44–1.93)	0.11 (0.04–0.33)	41.7% (33.9%–78.1%)	95.7% (87.8%–96.8%)
Reference Standard for EPS	78.6% (63.2%–89.7%)	59.6% (52.0%–66.8%)	1.94 (1.53–2.47)	0.36 (0.20–0.65)	31.4% (25.2%–52.1%)	92.2% (84.6%–94.2%)

CI, Confidence interval; EPS, Epigastric pain syndrome; FD, Functional dyspepsia; PDS, Postprandial distress syndrome

### Diagnostic performance of the Reference Standard symptom subtype modules

The performance of the Reference Standard for PDS and EPS was also compared against corresponding criteria in the Rome IV criteria. Among the sixty-six patients judged to have PDS based on the Rome IV criteria, sixty-three met the Reference Standard for PDS (Table 6), illustrating a sensitivity of 95.5% (95% CI 87.3%–99.1%) (Table 7). There were 154 individuals without Rome IV-defined PDS, of whom sixty-six did not meet the Reference Standard, showing a specificity of 42.9% (95% CI 34.9%–51.1%). The positive LR for PDS was 1.67 (95% CI 1.44–1.93), and the negative LR was 0.11 (95% CI 0.04–0.33). The AUC for the Reference Standard was 0.69 (95% CI 0.62–0.76).

Among the forty-two patients with a diagnosis of EPS under the Rome IV criteria, thirty-three met the Reference Standard for EPS (Table 6), demonstrating a sensitivity of 78.6% (95% CI 63.2%–89.7%) (Table 7). For the 178 patients who did not have Rome IV-defined EPS, 106 of them did not meet the Reference Standard as well, illustrating a specificity of 59.6% (95% CI 52.0–66.8). The positive LR for the Reference Standard for EPS was 1.94 (95% CI 1.53–2.47), and the negative LR was 0.36 (95% CI 0.20–0.65). The AUC for the Reference Standard was 0.69 (95% CI 0.64–0.74).

## Discussion

### Comparisons with similar studies

Since no previous studies were designed to investigate the diagnostic performance of the Reference Standard relative to the Rome IV criteria, we cannot compare the findings in this diagnostic study to those in other publications. That said, the Reference Standard has been evaluated against the Rome II criteria and the Rome III criteria for FD [12]. When compared against the Reference Standard, the Rome II criteria had a sensitivity of 71.4% (95% CI 68.4%–74.2%), a specificity of 55.6% (95% CI 51.5%–59.7%), a positive LR of 1.61 (95% CI 1.45–1.78), and a negative LR of 0.51 (95% CI 0.45–0.58). The Rome III criteria had a sensitivity of 60.7% (95% CI 57.5%–63.9%), a specificity of 68.7% (95% CI 64.6%–72.6%), a positive LR of 1.94 (95% CI 1.69–2.22), and a negative LR of 0.57 (95% CI 0.52–0.63).

### Implication for practice

This cross-sectional study illustrated that the Reference Standard may be sufficient in ruling out patients without Rome IV-defined FD, PDS, or EPS, given relatively high sensitivity values ranging from 78.6 to 95.5%. Their negative LRs of around 0.20 implied that negative test results of the Reference Standard may moderately decrease patients’ post-test probabilities of having FD, PDS, or EPS diagnosis [26]. However, affected by the mediocre specificity values ranging from 37.6 to 59.6%, the Reference Standard may not be useful in ruling in patients with Rome IV-defined FD, PDS, or EPS. The positive LRs of less than 2.0 also revealed that positive test results of the Reference Standard may only slightly increase patients’ post-test probabilities of having an FD, PDS, or EPS diagnosis [26]. The AUC values of around 0.70 indicated that the Reference Standard had moderate accuracy in distinguishing between patients with and without Rome IV-defined FD, PDS, or EPS [27].

With its satisfactory performance in ruling out patients without FD, PDS, or EPS, the Reference Standard may reduce unnecessary initiation of FD treatments. Reduction of over-treatment may reduce treatment-associated adverse events. The use of prokinetics, the recommended first-line therapy for FD in the Asian guideline [8], is associated with adverse events of dystonia, parkinsonism-type movements, tardive dyskinesia, or even life-threatening arrhythmia [9, 28]. Proton pump inhibitors, the first-line therapy for FD in the North American guideline [9], may increase the risk of hip fracture, community-acquired pneumonia, and Clostridium difficile infection [29]. Also, second-line therapy, tricyclic antidepressants, may cause constipation, dry mouth, urinary retention, and somnolence [9, 30].

By ruling out FD effectively, it is expected that the financial burden on FD patients and healthcare systems would be relieved by minimising the chance of initiating unnecessary treatments. A study in 2013 estimated that each FD patient in the United States had to pay, on average, USD805 per year for regular consultations and treatment [31]. These calculations did not consider the indirect cost incurred by absence from work and loss of productivity. A retrospective study in Malaysia also showed that FD is associated with the highest healthcare burden compared to other functional GI disorders in secondary care [32].

In routine practice where consultation time is limited, the Reference Standard may be used as an initial screening tool for FD and FD symptom subtypes prior to confirmation by the Rome IV criteria. If service arrangement allows, the Rome IV criteria should be implemented to confirm the positive results made by the Reference Standard to avoid potential false-positive cases, given the mediocre specificity of the former instrument. Moreover, the Rome IV criteria may be adopted for confirming FD diagnosis among patients who present with persistent dyspepsia symptoms but indeed show negative test results in Reference Standard screenings, facilitating appropriate initiation of treatment. Besides the questionnaire-based criteria, physicians should consider additional patient information [5, 33], such as symptom duration and co-morbidity, when making diagnoses for dyspeptic patients, since patients who do not fully meet the Rome IV criteria may still be offered essential treatments for reducing symptoms and improving quality of life [5, 8]. Additional diagnostic workup may also supplement OGD and H. Pylori tests for differential diagnosis. For example, in areas with a high prevalence of hepatocellular carcinoma like Southern China, upper abdominal ultrasound may be valuable for differentiating epigastric pain caused by malignancy or FD [8].

### Implication for research

Although the Rome diagnostic criteria were recognised in the Asian and North American FD guidelines for clinical research [8, 9], they were considered to have limited relevance to routine practice. To prepare for future updates, future research can investigate the feasibility of developing a concise edition of the Rome FD diagnostic criteria based on the Reference Standard, so as to facilitate FD diagnosis in outpatient clinics where consultation time is limited.

Furthermore, to introduce objectivity of FD diagnosis, the potential value of adding duodenal eosinophilia as a diagnostic marker should be investigated, since the phenomenon is closely associated with early satiety and PDS [34]. Gastric emptying is associated with the pathophysiological mechanism of FD [1, 28], so accelerated gastric emptying, delayed emptying, and fasting gastric volume may also be evaluated as potential motility markers for FD diagnosis [28, 35]. Alteration of the GI microbiota may also be chosen as another biomarker [28, 36], given its relationship with the occurrence of functional GI disorders.

Lastly, further diagnostic research may be conducted to investigate whether the Reference Standard is able to differentiate organic dyspepsia from FD. Evidence produced may contribute to reducing unnecessary tests and examinations in routine practice [13].

### Strengths and limitations

To the best of our knowledge, this study is the first to translate the Rome IV criteria for FD from English to Cantonese using the standardised forward–backward translation method. We held cognitive debriefing sessions with FD patients to test the clarity, adequacy of cultural adaptation, language usage, and acceptability of the draft translation. Each step in the translation process was monitored and approved by the official Rome Foundation. This study is also the first to compare the Reference Standard against the Rome criteria in terms of diagnostic performance and the first that is conducted in the China Greater Bay Area. Most importantly, this cross-sectional diagnostic study has low risk of bias and concerns over applicability in terms of the four domains in the QUADAS-2. These domains include: (i) patient selection; (ii) index test; (iii) reference standard; and (iv) flow and timing [17].

This study had certain limitations. First, given that only participants in Hong Kong were recruited for cognitive debriefing, local adaptations may be required before adopting the Cantonese R4DQ-FD in other Cantonese-speaking populations in China or overseas. Second, misclassifications of organic upper GI diseases might exist, since OGD and H. Pylori tests within five years were accepted for eligibility screening instead of referring the patient to concurrent diagnostic workup. Third, results from abdominal ultrasound were not included in the eligibility criteria, because the clinical value of the procedure in evaluating organic dyspepsia is limited [37]. Fourth, we used the prevalence of FD in Asia (30%) [38] for the sample size calculation because no information regarding the prevalence of FD in the China Greater Bay Area is available. This diagnostic study would have required a larger sample size if the prevalence of FD in the China Greater Bay Area was, in fact, lower than in Asia. Also, due to the lack of objective gold standard definitions of FD, the prevalence of FD used in this study may not reflect the true prevalence of FD in the continent. Fifth, the diagnostic performance indicators of a diagnostic test may vary between subgroups of participants with different demographical or clinical characteristics [39]. These characteristics may include but are not limited to age, gender, and symptoms’ severity and frequency [39]. However, due to the relatively small sample size, we did not conduct logistic regression analyses to explore the relationships between the diagnostic performance indicators of the Reference Standard and participant subgroups.

## Conclusions

Given relatively high sensitivity values, the Reference Standard is capable of ruling out patients without FD, PDS, or EPS. It can be used as a concise instrument for FD initial screening in routine practice, especially in settings where the use of the Rome IV diagnostic instrument is not practical. The Reference Standard may also serve as a uniform FD diagnostic instrument for FD in future updates of clinical guidelines. Future research should focus on establishing FD diagnostic criteria based on the Rome criteria with higher practicality, objectivity, and population relevance.

## Supplementary Information

**Additional file 1**. **Appendix 1.** Translation of the Rome IV diagnostic questionnaire for functional dyspepsia; **Appendix 2.** Formulae for calculating diagnostic performance indicators.

## Data Availability

The datasets generated during and/or analysed during the current study are available from the corresponding author on reasonable request.
