# Supplementary material for: Comparing diagnostic performance of Cantonese-Chinese version of Rome IV criteria and a short Reference Standard for functional dyspepsia in China
Source: BMC Gastroenterol. 2022 Oct 12;22:432. doi: 10.1186/s12876-022-02520-6 (PMC9558384; doi:10.1186/s12876-022-02520-6)
Supplement: Supplementary file 1 — Additional file 1. Appendix 1. Translation of the Rome IV diagnostic questionnaire for functional dyspepsia; Appendix 2. Formulae for calculating diagnostic performance indicators. [file 12876_2022_2520_MOESM1_ESM.docx]

**Supplementary file**

**Appendix 1.** Translation of the Rome IV diagnostic questionnaire for functional dyspepsia

Step 1: Forward translation – the original Rome IV diagnostic questionnaire for functional dyspepsia (R4DQ-FD) was translated into Cantonese-Chinese (Cantonese) by two professional medical translators who were knowledgeable about health terminology, as well as cultural and linguistic nuances of Cantonese. Two independent drafts of Cantonese R4DQ-FD were produced.

Step 2: Reconciliation – a reconciliation process was conducted by an experienced gastroenterologist (JCYW) and the two translators to compare the two Cantonese translations. This process allowed the identification of ambiguities and discrepancies in wording, sentence structure, meaning, and response options. One single preliminary Cantonese R4DQ-FD was generated in this process.

Step 3: Backward translation – Another professional medical translator with English as the mother language translated the preliminary Cantonese R4DQ-FD back into English. A back-translated R4DQ-FD was created.

Step 4: Comparison of the English versions – a committee consisting of the gastroenterologist and the three translators compared the back-translated R4DQ-FD with its original English version. The comparison focused on wording, sentence structure, meaning, and response options. Discrepancies between the two versions were identified and were used for amending wording choices in the preliminary Cantonese R4DQ-FD. These amendments generated the pre-final Cantonese R4DQ-FD for further cognitive debriefing.

Step 5: Cognitive debriefing – ten FD patients were invited to individual cognitive debriefing sessions. In each session, they were invited to evaluate the clarity, adequacy of cultural adaptation, language usage, and acceptability of the pre-final Cantonese R4DQ-FD. They were also invited to suggest necessary language amendments. Finally, after considering patients’ feedback, minor adjustments were made to the pre-final R4DQ-FD by the translation project lead (VCHC) and the committee convened for the previous step. The final Cantonese R4DQ-FD was produced for further assessment of diagnostic performance.

**Appendix 2.** Formulae for calculating diagnostic performance indicators

|  |  | **Rome IV criteria** | | |
| --- | --- | --- | --- | --- |
|  |  | Positive | Negative | Total |
| **Reference Standard** | Positive | a | b | a + b |
|  | Negative | c | d | c + d |
|  | Total | a + c | b + d | a + b + c + d |

| Sensitivity of the Reference Standard:  $\frac{a}{a+c}\times100\%$ |
| --- |
| Specificity of the Reference Standard:  $\frac{d}{b+d}\times100\%$ |
| Positive likelihood ratio of the Reference Standard:  $\frac{Sensitivity of the Reference Standard}{(100\%-Specificity of the Reference Standard)}$ |
| Negative likelihood ratio of the Reference Standard:  $\frac{(100\%-Sensitivity of the Reference Standard)}{Specificity of the Reference Standard}$ |
| Positive predictive value of the Reference Standard:  $\frac{a}{a+b}\times100\%$ |
| Negative predictive value of the Reference Standard:  $\frac{d}{c+d}\times100\%$ |

FD: Functional dyspepsia.
